# The Expansion of the Horse’s Foot When Shod and Unshod

**Published:** 1881-10

**Authors:** John N. Navin

**Affiliations:** Veterinary Editor of Indiana Farmer


### ART. XVII.—THE EXPANSION OF THE HORSE'S FOOT WHEN SHOD AND UNSHOD.

### PREFATORY REMARKS.

Horseshoeing was first practiced by the Arabs, and from that time until recently the necessity for protection to the foot from wear and tear has seldom been doubted by sensible men in any country, the only difference of opinion having existed in the form of shoe, and in the manner of fastening to the foot.

The first shoe used seems to have been nothing more than an oval piece of sheet iron with a hole in its centre; but its form had soon been changed and made square at the heels. Both forms, however, seem to have been fastened on by some device other than by nailing, from the fact that the holes representing the nail-holes of the present shoe in use were buttonhole, or longitudinal shaped from within outward toward the edge.

It also appears that the English were the first to adopt shoes open at the heel and centre of foot, and nailed as at present. This seems to have been the first material improvement, though the Moors, the Persians, and Portuguese had priorly made important changes from the Arabian pattern. For a long time very little improvement had been made, but within the last half century horseshoeing has attracted almost universal attention, as well among the ablest veterinarians as among a class of men calling themselves veterinary surgeons, but who possess neither theory or practical skill—enthusiasts, such as are found among all professions. The veterinary profession, however, seems to suffer more from quackery than all others combined, for every town, city and country vicinity are cursed by this class of men, who are, in fact, more dangerous than disease is to the horse. If advised to study the profession, they become very indignant, and tell you they have been raised with and among horses, have used them all their life-time, therefore know all about them and their diseases, and especially are they posted, as they term it, in regard to the foot; and from time to time have invented the most senseless theories and forms of shoe; while others of the same class have contended that the horse could have been worked and ridden upon the hardest and roughest roads unshod without injury.

But all such senseless theories, and as many forms of shoe, have given way to the old concave-seated shoe, whose only objective feature is the nailing down of an elastic foot to an unyielding piece of iron called a shoe, with four stout nails on each side which prevent its natural expansion and contraction, and is the cause of all the evils incident to the shod foot; therefore shoeing in the past has been no more or less than a necessary evil.

From the earliest date in shoeing to the present, it appears that every man of judgment who paid proper attention to the matter, saw and admitted that the foot is an elastic pedal organ, expanding when pressed on and resuming its normal form when relieved from such pressure, and that any form of shoe which impeded its expansion and contraction must in some way be detrimental to its healthy condition. Indeed, this idea must force itself upon the most obtuse mind when we consider the admitted fact that, except by accident, the colt's foot is free from contraction, corns, and other evils until being shod.

Scarcely is a man to be met now-a-days, who pretends to anv knowledge of the horse, who is ignorant enough to not admit the above facts, and to that end scores, if not hundreds, of different formed shoes have been devised, and for a time used, but soon laid aside as useless for the purpose they were intended, all having failed to permit the foot to expand and contract in violent action, while others proved directly injurious by their first application.

The mode of fastening the different formed shoes to the foot has been and still is, perhaps, the greatest difficulty in safe shoeing. Still, in both the shape and manner of fastening, every inventor of horseshoes, except, perhaps, two, have had an idea that the foot should be permitted to expand when pressed upon, and to contract as soon as relieved of such pressure. One of the last named fastened his shoe with clips turned up at the toe and sides of the foot. Of course, no foot could have long endured such pressure, so it was promptly thrown into disuse; another bright genius fastened his shoe on with wire bands, and shared a similar short existence. Indeed, quacks are not alone responsible, for many of the profession are found among the inventors of such devices of torture.

Every veterinarian of respectable standing in the profession is competent to intelligently name the different organs composing the foot; but this is not all sufficient; we must also know and be able to explain the relation of each to the others as a whole, and especially to the organs with which they are in contact; how each organ is produced, and how reproduced whenever destroyed by wear and tear or accident. That is to say, all which are susceptible of being produced and reproduced by others. This knowledge we must possess before we are competent to apply a shoe to the foot that is not, as heretofore, a necessary evil.

And although the idea that a shoe which permitted the foot to expand and contract had been impressed upon the minds of the inventors of shoes by the force of theory set forth in books by able writers, it brought no relief to the dumb sufferer from malpractice, if I may be allowed the appellation for bad shoeing. For men still ignorant of nature's adopted means for the expansion of the foot got it into their minds that frog pressure was the only means of expansion by coming in contact with the ground, therefore nine-tenths of all the foolish devices in shoeing have been based upon the theory of frog pressure ; and, indeed, many learned veterinary surgeons have been fooled into the idea, and had their force of thought unfortunately turned in that direction. But these, on failure to provide a suitable shoe, wisely used the old concave-seated shoe as the least evil and last resort

Therefore, inexperienced men went to work to make frog pressure shoes. The object in their application was to lower the ground surface of the shoes to a level with the base of the frog, hoping that its pressure against the ground would so expand the wall as to permit the sole to yield under the pressure of the coffin bone upon its internal convex crown in violent action, not possessing sufficient anatomical knowledge to see that the frog is not in full contact with the wall, therefore cannot act as a wedge sufficient to force it apart. Secondly, the shoe being broader than the wall is thick, would not permit the frog to reach it, so there is an end to frog pressure with sunken shoes.

"Oh, but," its advocates say, "the frog presses the sole, and the sole the wall." Indeed; let us see if this is possible. The sole, bars, and wall are cut down in order to let the ground surface of the shoe down to a level with the base of the frog. So there is neither sole left to press against the wall, nor wall to be pressed by the sole, for both are cut away so low that a little more cutting would open into the sensitive parts of the foot.

So much for frog pressure by cutting down the parts. Another frog pressure scheme had been tried with tips, which consists in a shoe reaching from the toe to near the quarters. This proved no better than the former, for it elevated the toe, and permitted the heels to wear down. This shoe threw too much strain upon the tendons and ligaments, and again the concave seated shoe gained a victory.

In the near future, I propose to prove that nature has provided means for the expansion of the foot, independent of frog pressure. That the unshod foot expands independent of it; that the old method of nailing the foot to an unyielding shoe has been the cause of all the evils consequent upon shoeing in the past; and I also propose to furnish a shoe the application of which permits the full and free expansion of the foot when violently pressed, and its contraction as soon as relieved of such pressure, as readily as does the colt's unshod foot. It is now in use four months, and has cured every horse shod with it of contraction, corns, and other maladies, and it is impossible for any horse wearing it to become lame, except by accident.

JOHN N. NAVIN, V.S.,
*Veterinary Editor of Indiana Farmer.*

(*To be continued.*)

